# High Foetal Haemoglobin in Sickle Cell Disease: Not so Protective?

**DOI:** 10.1016/j.ebiom.2015.01.005

**Published:** 2015-01-14

**Authors:** Nicola Conran

**Affiliations:** INCT de Sangue, Hematology Center, School of Medicine, University of Campinas — UNICAMP, Campinas, Sao Paulo, Brazil

Sickle cell disease (SCD) comprises a group of genetic disorders in which the red blood cells (RBCs) produce abnormal sickle haemoglobin (HbS) that can polymerise when oxygen concentrations are low. The clinical manifestations of SCD are numerous, and vary from patient to patient, but recurrent vaso-occlusive processes can cause significant organ damage, resulting in increased morbidity and mortality in these individuals (Kato et al., 2009). Polymerised HbS confers a characteristic sickle shape to the RBC, in association with other cellular alterations; furthermore, these RBCs are more likely to rupture, releasing damaging cell-free haemoglobin (Hb) into the circulation (haemolysis), with significant consequences that include vascular nitric oxide (NO) consumption and oxidative stress (Kato et al., 2009). Newborn and very young infants with sickle cell anaemia (SCA) still produce significant amounts of foetal haemoglobin (HbF), the Hb that is made during intrauterine life, although this HbF production will begin to decline after the first few months due to the Hb-switching process (Quinn, 2013). As high concentrations of HbF inhibit the polymerisation of HbS, young infants are asymptomatic and are generally thought to display little in the way of pathophysiological alterations.

In the current issue of E-BioMedicine, Brousse et al. (in press) report intriguing data to show that very young asymptomatic SCA infants, whilst still producing very high total levels of HbF, demonstrate significant alterations in reticulocyte membrane protein expression, in association with reticulocytosis. The infants with SCA studied by Brousse and colleagues were aged approximately 3–6 months and presented total HbF levels of 41.2 (± 11.2) %, a level much higher than that generally sought during HbF-inducing hydroxycarbamide (hydroxyurea) therapy in SCA adults (McGann and Ware, 2011). However, despite presenting levels of total HbF thought to be protective in SCA, the adhesion molecule expression profile on the reticulocytes of these children was altered. Changes in adhesion molecule presentation on the erythroid surface can confer alterations in cellular adhesion and augmented RBC adhesive properties have been consistently observed in SCA, where erythroid cell adhesive interactions are believed to contribute to the initiation and propagation of vaso-occlusive processes (Colin et al., 2014). As such, the alterations observed in the expression profile of major adhesion molecules on the reticulocyte surface of young infants could have some important pathophysiological consequences; splenic vaso-occlusion and the onset of hyposplenism, for example, usually occur before one year of age in SCA and the adhesion of reticulocytes to the splenic vessels from a very early age could participate in this mechanism (Brousse et al., 2014, Brousse et al., in press). Furthermore, although longitudinal data are required for confirmation, the circulation of stress reticulocytes and reduced Hb levels observed in these infants could be suggestive of haemolytic anaemia. Given the known hazards of haemolysis (Kato et al., 2009), it would seem of importance to characterise these events further in young infants with a view to determining whether clinical approaches to limit haemolysis should considered even before the emergence of symptomology.

The distribution of HbF among HbF-containing red cells (F-cells) varies between SCA individuals (Horiuchi et al., 1995), and this study also highlights recent suggestions that the distribution of HbF among red cells is more important than the total amount of HbF produced, in terms of its protective effects (Steinberg et al., 2014), as the concentration of HbF in each F-cell must be sufficient to interfere in HbS polymerisation and therefore reduce the symptoms of SCA (Steinberg et al., 2014). Co-inheritance of hereditary persistence of HbF (HPFH) together with HbS, for example, typically induces levels of approximately 30 % HbF in patients; this HbF, however, is usually distributed among all the red cells and, consequently, these individuals are asymptomatic (Steinberg et al., 2014, Ngo et al., 2012). In infants with SCA, the mean HbF/F-cell content is known to drop drastically during the first year of life, whilst the percentage of HbF cells remain high (Marcus and Ware, 1999), although the distribution of HbF concentrations in the F-cells is not known. Thus, Brousse and colleagues' observation that very high levels of global HbF did not completely protect young infants from red cell alterations and reticulocytosis could indicate the occurrence of a heterocellular distribution of HbF in these patients, whereby inhibition of HbS polymerisation may not occur in a subpopulation of erythroid cells. Whilst the success of hydroxycarbamide as a therapy for SCA is undeniable, a growing body of evidence indicates that its extensive clinical benefits may lie not only in its ability to elevate HbF, but may also occur via HbF-independent mechanisms (McGann and Ware, 2011). Researchers in the field continue to develop drugs and approaches that focus on elevating HbF; while the importance of augmenting HbF in SCA is irrefutable, care should be taken to ensure that HbF is elevated in a pan-cellular manner and that combination drug approaches are used to target other aspects of SCA pathophysiology.

## Conflicts of Interest

The author declares no conflicts of interest relevant to this commentary.
